# Mandibular involvement in SAPHO syndrome: a retrospective study

**DOI:** 10.1186/s13023-020-01589-0

**Published:** 2020-11-05

**Authors:** Mu Wang, Yueting Li, Yihan Cao, Xinyu Lu, Yuchen Liu, Jizhi Zhao, Wen Zhang, Chen Li

**Affiliations:** 1 Department of Stomatology, Peking Union Medical College Hospital, Peking Union Medical College, Chinese Academy of Medical Sciences, Beijing, 100730 China; 2 Department of Traditional Chinese Medicine, Peking Union Medical College Hospital, Peking Union Medical College, Chinese Academy of Medical Sciences, Beijing, 100730 China; 3 Department of Rheumatology and Clinical Immunology, Peking Union Medical College Hospital, Clinical Immunology Center, Peking Union Medical College, Chinese Academy of Medical Sciences, Beijing, 100730 China; 4 Department of Radiology, Peking Union Medical College Hospital, Peking Union Medical College, Chinese Academy of Medical Sciences, Beijing, 100730 China; 5 Peking Union Medical College, Chinese Academy of Medical Sciences, Beijing, 100730 China

**Keywords:** Mandible, Osteitis, Cone-beam computed tomography, Magnetic resonance imaging, Diphosphonates, Etanercept

## Abstract

**Background:**

Mandible osteomyelitis can occur in synovitis, acne, pustulosis, hyperostosis, and osteitis (SAPHO) syndrome, a rare chronic inflammatory disease; however, few studies have explored its characteristics and management.

**Methods:**

We reviewed the medical records of consecutive SAPHO patients with mandible involvement diagnosed in Peking Union Medical College Hospital from September 2014 to July 2019. Demographic, clinical, laboratory, and imaging data were collected at baseline. Prescription data and follow-up magnetic resonance imaging (MRI) and cone beam computed tomography (CBCT) images were collected from the hospital information system. An electronic questionnaire was distributed to all patients to obtain their latest symptoms.

**Results:**

A total of 26 SAPHO patients with mandibular involvement were involved, all of whom responded to the questionnaire (38.5% male; median age, 28 years; median follow-up duration, 2.1 years). Ten patients (38.5%) had undergone an oral procedure 1 month before the onset of mandibular symptoms. All 14 of the patients who underwent a surgical intervention relapsed within a median duration of 2 months (range 0.25–4.0 months), and 24 patients (92.3%) achieved improvement with conservative treatment. Following bisphosphonate treatment, remission of bone marrow oedema and osteolysis was observed on MRI and CBCT, and 5 patients receiving bisphosphonates with follow-up CBCT after remission did not relapse in 5.4 months (mean 6.0, range 3.2–9.9 months).

**Conclusion:**

Mandibular involvement of SAPHO syndrome predominantly occurs in young women. Dental procedures are a possible risk factor. Conservative treatment, especially intravenous bisphosphonates, can lead to oral improvement.

## Introduction

Synovitis, acne, pustulosis, hyperostosis, and osteitis (SAPHO) syndrome is a chronic inflammatory disease characterized by osteoarticular involvement with or without palmoplantar pustulosis (PPP) or severe acne (SA) [1]. This rare disease has an annual prevalence of 0.00144/100,000 to 1/10,000 [2, 3], and it imposes a heavy burden on the health and quality of life of affected patients [4]. SAPHO syndrome can occur at any age, but predominantly affects female patients [5–7]. SAPHO aetiology remains elusive and may be associated with genetic, infectious, and autoimmune factors [3].

Osteoarticular disorder is the characteristic feature of SAPHO syndrome and typically involves the anterior chest wall. The peripheral bones are affected in one-third of patients [3, 5]. Mandibular lesions occur in 2–10% of SAPHO patients and are generally located on the posterior mandibular body and ramus [8, 9]. Few patients present with involvement of the temporomandibular joint (TMJ), which is considered an extension of mandible osteitis [8]. Radiologically, mandible lesions present initially as osteolysis and an associated periosteal reaction, resulting in sclerosis and hyperostosis [10, 11]. Patients commonly visit an oral and maxillofacial surgeon due to obvious swelling and pain in the jaw and typically receive a decortication or partial mandible resection, which has a controversial prognosis [12, 13]. Treatment with medications, including bisphosphonates and tumour necrosis factor α inhibitors (TNF inhibitors), have been used and clinically demonstrated to be effective in SAPHO syndrome [14–16]; however, no large cohort study of SAPHO patients with mandibular involvement or detailed long-term radiological follow-up of their mandible lesions has been performed to confirm treatment efficacy.

Here, we present our follow-up cohort of 26 SAPHO patients with mandible involvement and report their epidemiology, clinical and laboratory features, whole body scintigraphy manifestations, and follow-up mandible lesions found on cone beam computed tomography (CBCT) and magnetic resonance imaging (MRI).

## Methods

### Patients

**Fig. 1:**
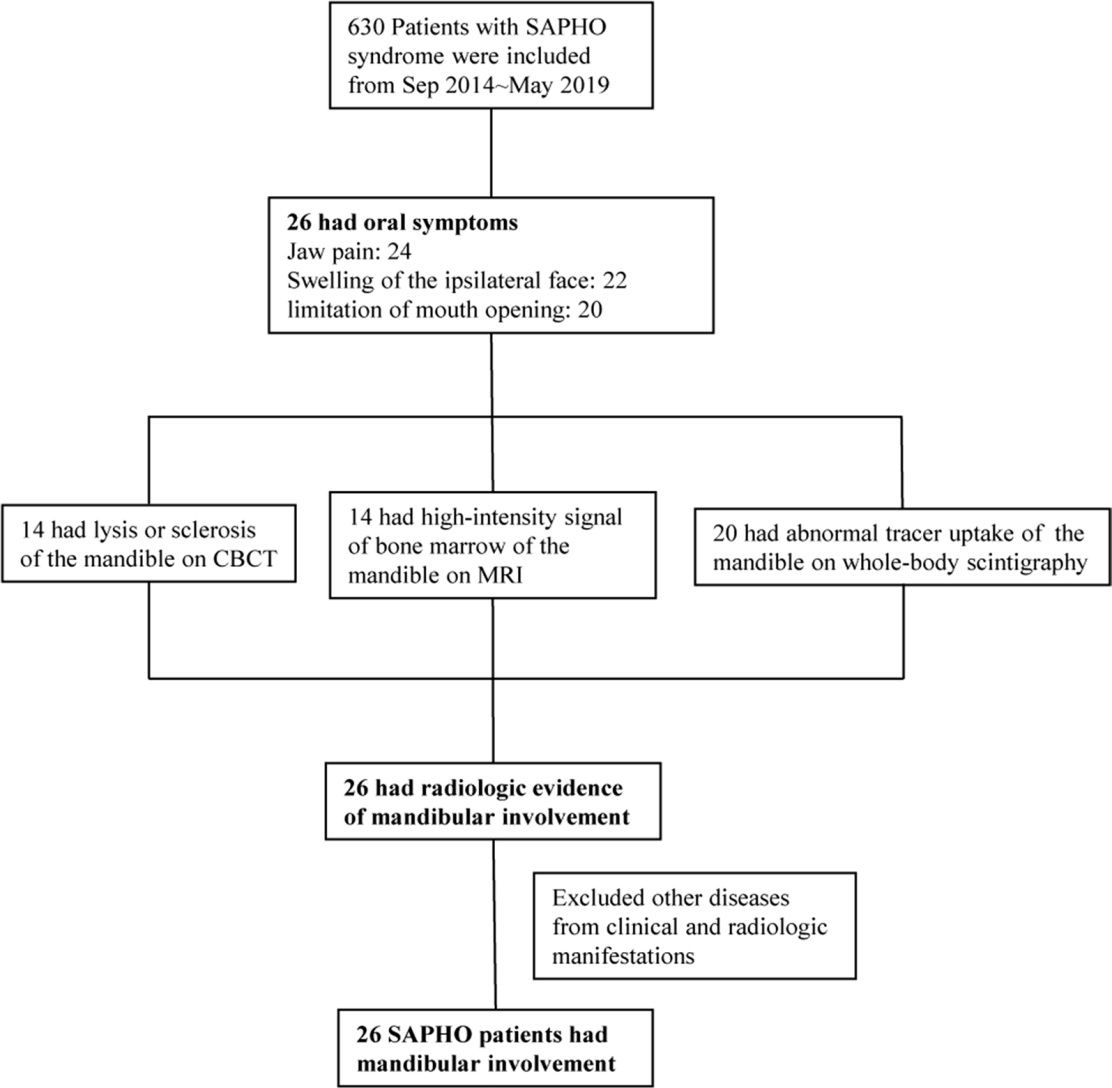
Flowchart of inclusion and exclusion criteria

Patients who met the criteria for SAPHO syndrome proposed by Kahn MF (2003 ACR 67th Annual Scientific Meeting) were recruited in Peking Union Medical College Hospital [17]. Two rheumatologists, CL and WZ, diagnosed SAPHO syndrome; two stomatologists, MW and JZ, confirmed involvement of the mandible in consensus. We retrospectively reviewed the medical records to identify patients with mandibular involvement from September 2014 to July 2019 (Fig. 1). Mandibular involvement of SAPHO patients was confirmed based on the following: (1) clinical symptoms and signs: swelling and pain of the mandible area; enlargement of the mandible; (2) radiologic or pathologic evidence: abnormality in the mandibular region on whole-body scintigraphy, CBCT, or MRI; or non-inflammatory osteitis on biopsy; and (3) exclusion of other disease (infectious osteitis, tumour, etc.). This study complied with the Declaration of Helsinki and was approved by the Ethics Committee of Peking Union Medical College Hospital. Written informed consent was obtained from each patient.

### Clinical, biological, and imaging data

We retrospectively reviewed demographic data, including age, sex, time of onset, and experience of dental procedure within 1 month before the onset of oral symptoms, obtained at the first visit. Dental procedures included root canal therapy, tooth extraction, dental implantation, orthodontic treatment, and gingival resection. We also collected clinical manifestations of the skin and musculoskeletal system, a medical history of drug and surgery treatment, and the visual analogue scale (VAS) scores recorded at baseline. The levels of inflammatory markers, including high-sensitivity C-reactive protein (hs-CRP) and ESR, and human leukocyte antigen (HLA) B27, were measured. Whole-body bone scintigraphy was performed using ^99m^Tc-MDP in all patients, and abnormal tracer uptake linked to SAPHO syndrome was recorded in detail. In some patients, further treatment assessment included panoramic dental X-rays, CBCT imaging and 1.5-T MRI of the maxillofacial region. In imaging studies, progression on CBCT was defined as a new or increased area of cortical or medullary lysis, and progression on MRI was defined as a new or increased area or increased intensity of the high-intensity signal of bone marrow or surrounding soft tissue on the T2-weighted image. Remission on CBCT was defined as a decreased area of cortical or medullary lysis, and remission on MRI was defined as a decreased area or intensity of the high-intensity signal of bone marrow or surrounding soft tissue on the T2-weighted image. The healthy part of the mandible was used as a reference.

### Follow-up

We collected clinical, laboratory, radiologic, and prescription data from the hospital records of every visit at PUMCH. We distributed an electronic questionnaire to all patients in May 2019 to obtain their latest medical compliance and improvement in symptoms. For patients with follow-up MR or CBCT images, the use of drugs between every two MR or CBCT scans was listed. Any changes in SAPHO-related lesions as observed on MR images after treatment were also recorded.

### Statistical analysis

Data analysis was conducted using SPSS software, version 24 (SPSS Inc, Chicago, Illinois, USA). Descriptive data shown as the median (interquartile ranges [IQR]), or median (range), or a number (%). χ^2^ and Fisher’s exact tests were used to compare categorical data expressed as numbers(percentages) of subjects. Mann–Whitney *U *test was used to compare continuous data summarized as medians and interquartile ranges (IQR) or ranges. All tests were two-tailed with a significance level of 0.05.

## Results

### Demographic features

A total of 26 patients were included in our study (Fig. 1). The 10 male and 16 female patients had a median age of 28 years (range 5–65 years) (Table 1) and were younger than SAPHO patients without mandibular involvement (28 vs. 40 years, *p* < 0.001). The median age at SAPHO syndrome onset of patients with mandibular involvement was younger than that of patients without mandibular involvement (22 vs. 37 years, *p* < 0.001). The median age at the onset of related oral symptoms was 25 years (IQR 15–28 years). Ten patients (38.5%) had received a dental procedure within 1 month before the onset of their oral symptoms, eight had teeth extractions, four had root canal therapy, and one had gingival resection.

**Table 1:** Demographic and clinical characteristics of the patients with mandibular involvement of SAPHO syndrome

Variables	Mandibular involvement		*p* value^b^
	+ (n = 26)	− (n = 604)	
Age (years), median (range)	28 (5–65)	40 (10–71)	< *0.001*
Sex (males/females), n (%)	10/16 (38.5/61.5)	193/411 (32.0/68.0)	0.522
Disease duration at baseline (years), median (IQR)	2 (1–8)	2 (0.6–5)	*0.011*
Age at onset of symptoms (years), median (IQR)	22 (14–27)	37 (29–48)	< *0.001*
Age at onset of oral symptoms (years), median (IQR)	25 (15–28)	–	–
Duration of diagnosis (years), median (IQR)	2 (0.8–3.0)	2 (0.7–4.7)	0.291
Dental procedure^a^ in 1 month before the onset of oral symptoms, n (%)	10 (38.5)	–	–
Affected region of the mandible		–	–
Left side, n (%)	7 (26.9)	–	–
Right side, n (%)	10 (38.5)	–	–
Bilateral, n (%)	9 (34.6)	–	–
Oral symptoms			
Jaw pain, n (%)	24 (92.3)	–	–
Swelling of the ipsilateral face, n (%)	22 (84.6)	–	–
Limitation of mouth opening, n (%)	20 (76.9)	–	–
Skin manifestations, n (%)	15 (57.7)	531 (87.9)	< *0.001*
PPP, n (%)	8 (30.1)	490 (81.8)	< *0.001*
SA, n (%)	7 (26.9)	61 (10.1)	*0.024*
VAS, median (IQR)	5 (3–6)	4 (3–6)	0.311
Serum hs-CRP (mg/ml), median (IQR)	6.41 (2.39–21.59)	4.00 (1.95–9.03)	0.641
ESR (mm/h), median (IQR)	21 (14–31)	14 (8–28)	0.413
HLA-B27 positive, n (%)	0 (0)	27 (4.7)	0.623

*IQR* interquartile ranges, *BMI* body mass index, *SA* severe acne, *PPP* palmoplantar pustulosis, *VAS* Visual Analogue Scale, *ESR* erythrocyte sedimentation rate, *hs-CRP* high-sensitivity C-reactive protein, *HLA* human leukocyte antigen

^a^Dental procedure: root canal therapy, teeth extraction, dental implant, orthodontic treatment, and gingival resection

^b^All the tests were two-tailed with a significance level of 0.05

### Clinical, laboratory, and radiologic characteristics at baseline

Seventeen patients (65.4%) showed unilateral mandibular involvement; of the other nine patients, eight had unilateral mandibular involvement at onset (Table 1). Jaw pain, swelling of the ipsilateral face, and limitation of mouth opening occurred in 24 (92.3%), 22 (84.6%), and 20 (76.9%) patients, respectively. Skin disorders affected 15 patients (57.7%) with mandibular involvement, fewer than patients without mandibular involvement (57.7% vs. 87.9%, *p* < 0.001). Patients with mandibular involvement had less palmoplantar pustulosis (PPP) (30.1% vs. 81.8%, respectively, *p* < 0.001) and more severe acne (SA) (26.9% vs. 10.1%, respectively, *p* = 0.024). The median VAS score was 5 (IQR 3–6). No patient had a positive HLA-B27 test.

Whole-body scintigraphy showed anterior chest wall involvement in 9 patients (36.0%), axial skeletal involvement (spine or sacroiliac joints) in 8 (32.0%), and peripheral osteoarticular involvement in 9 (36.0%) (Additional file 1: Table S1). Among the affected peripheral joints, large joints including the shoulder (1, 4.0%), knee (3, 12.0%), and ankle (1, 4.0%) were most commonly affected.

**Fig. 2:**
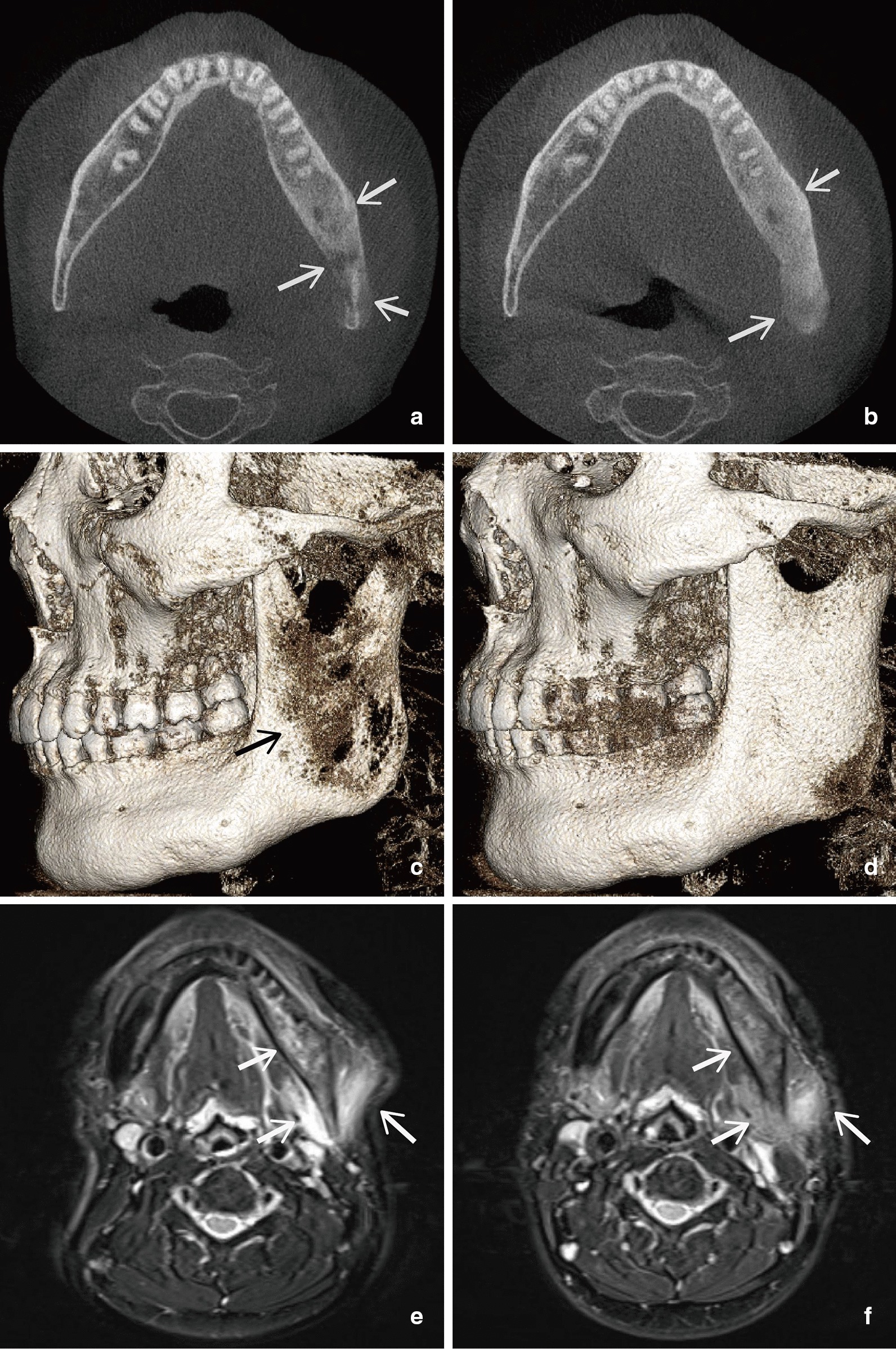
Mandible CBCT axial view and 3D reconstruction of a TNF-α inhibitor resistant patient (**a**, **c**) at baseline, showing diffuse lysis, sclerosis, and subperiosteal bone formation (**b**, **d**), and 1 year after bisphosphonate treatment, showing mainly sclerosis and hyperostosis. Fat Suppression T2-Weighted MR imaging (**e**) at baseline, showing oedema of bone marrow and masticatory muscles, and **f** 1 year after bisphosphonate treatment, showing alleviation of oedema

Fourteen patients underwent CBCT scanning of the maxillofacial region in our hospital (Table 2, Fig. 2a, c). CBCT images were evaluated for osseous lesions of the cortex and medulla, including the width of the cortical bone, cortical lysis, medullary sclerosis and lysis, subperiosteal bone formation, and hyperostosis. Cortical bone thinning and cortical lysis were found in eleven (78.6%) and six (42.9%) patients, respectively. Medullary sclerosis and lysis appeared in eight (57.1%) and four (28.6%) patients, respectively. Subperiosteal bone formation also occurred in four patients (28.6%), and hyperostosis occurred in eight patients (57.1%).

**Table 2:** Radiographic findings in SAPHO patients with mandible involved at baseline (n = 16)

Radiographic findings	n (%)
CBCT (N = 14), n (%)	
Cortical bone thinning	11 (78.6)
Cortical lysis	6 (42.9)
Medullary sclerosis	8 (57.1)
Medullary lysis	4 (28.6)
Subperiosteal bone formation	4 (28.6)
Hyperostosis	8 (57.1)
MR (N = 14), n (%)	
Muscle edema	9 (64.3)
Massetter	9 (64.3)
Medial pterygoid muscle	7 (50.0)
Lateral pterygoid muscle	1 (7.1)
Subcutaneous soft tissue swelling	9 (64.3)
Bone marrow edema	14 (100.0)
Periostitis	6 (42.9)
Synovial thicknening of temporomandibular joints	3 (21.4)

*CBCT* cone beam computed tomography, *MR* magnetic resonance

Fourteen patients underwent MRI of the maxillofacial region (Table 2, Fig. 2E). MR images assessed muscle oedema, soft tissue swelling, medullary oedema, periostitis, and synovial thickening of the TMJ. Nine (64.3%) patients displayed muscle oedema, and of these, nine (64.3%) had oedema in the masseter, seven (50.0%) in the medial pterygoid muscle, and one (7.1%) in the lateral pterygoid muscle. Subcutaneous soft tissue swelling appeared in nine patients (64.3%), including seven with muscle oedema. All 14 patients (100%) had medullary oedema in the mandible, and six (42.9%) had periostitis. Synovium thickening of the TMJ was also recorded in three patients (21.4%).

### Treatment and follow-up

All patients were followed up in clinical interviews until May 2019, and all completed our questionnaire. The median follow-up duration was 2.1 (mean 2.1, range 0.1 to 6.0) years. Fourteen patients underwent a surgical intervention before the first visit to our hospital; twelve (46.1%) had decortication and two (7.7%) had resection of the unilateral mandible (Additional file 1: Table S2). All 14 patients relapsed before visiting our hospital, and the median duration between the surgery and relapse was 2.0 months (mean 1.8, range 0.25–4 months). Conservative treatment was reported to be effective in the alleviation of jaw pain in 24 (92.3%) patients (Additional file 1: Table S3). Reports of improvement in dermatological (13.3%) and other osteoarticular symptoms (37.5%) were less common.

Fourteen patients had follow-up CBCT images (Table 3, Fig. 2b, d). The median interval between CBCT scans was 5.4 months (range 1.9–26.4 months). All six patients who received bisphosphonates showed a decrease in the sizes of the areas showing cortical or medullary lysis, and two of the four patients who received a TNF inhibitor achieved remission. Neither corticosteroids alone nor the IL-6 inhibitor alone was effective in achieving bone lysis remission. Eight patients had follow-up CBCT after remission. Five patients receiving bisphosphonates did not relapse in a median duration of 5.4 months (median 6.0, range 3.2–9.9 months). Another two patients receiving bisphosphonates with TwFH or glucocorticoid did not relapse in 24.0 and 12.1 months, respectively. One patient continued methotrexate and glucocorticoid treatment and did not relapse in 15.6 months.

**Table 3:** CBCT cortical and medullary lysis change (N = 14) and MRI change (N = 14) in patients after treatment

Treatment^a^	CT			MRI										
	N	Remission^b^	Progression^c^	N	Muscle edema		Subcutaneous soft tissue swelling		Bone marrow edema		Periosteitis		Synovial thickening of TMJ	
					R	S/P	R	S/P	R	S/P	R	S/P	R	S/P
TNFi	4	2	2	3	1	1	0	1	0	3	0	1	0	0
TNFi + CS	1	0	1	0	–	–	–	–	–	–	–	–	–	–
TNFi + MTX	0	–	–	3	1	2	0	1	1	2	0	0	0	0
TNFi + TwFH	0	–	–	1	0	1	0	0	0	1	0	0	0	0
BP	6	6	0	5	3	1	1	0	4	1	1	0	1	0
BP + CS	1	1	0	0	–	–	–	–	–	–	–	–	–	–
BP + TwFH	1	1	0	1	0	0	0	0	0	1	0	0	1	0
BP + TNFi	2	2	0	1	1	0	0	0	1	0	0	0	0	0
TwFH + CS	0	–	–	4	4	0	1	0	2	2	1	1	3	0
CS	2	0	2	3	2	1	1	0	2	1	0	0	1	1
CS + MTX	1	1	0	0	–	–	–	–	–	–	–	–	–	–
IL-6 inhibitor	1	0	1	0	–	–	–	–	–	–	–	–	–	–

*R* remission, *S/P* stable disease/Progression, *TNFi* tumour necrosis factor-α inhibitor, *MTX* methotrexate, *TwFH* *Tripterygium wilfordii* Hook F, *CS* Corticosteroids, *TMJ* temporomandibular joint, *BP* bisphosphonate

^a^One patient could change the drug due to its uneffectiveness during the follow-up

^b^Remission: a new or increased area of cortical or medullary lysis on CBCT, or a new or increased area or increased intensity of the high-intensity signal of bone marrow or surrounding soft tissue on the T2-weighted image

^c^Progression: a decreased area of cortical or medullary lysis on CBCT, or a decreased area or intensity of the high-intensity signal of bone marrow or surrounding soft tissue on the T2-weighted image

Fourteen patients had at least one MRI scan during follow-up (Table 3, Fig. 2f). The median interval between MR scans was 5.5 months (range 3.0–22.0 months). Both the modulation of bone metabolism by bisphosphonates and the control of systemic inflammation using GS or TNF inhibitor were effective in some patients, including those treated with or without DMARDs or an immunosuppressive drug, *Tripterygium wilfordii* Hook F (TwHF) [18]. Bone marrow oedema remission was observed in four of the five patients (80%) who received bisphosphonate, including two in whom a TNF inhibitor was ineffective.

## Discussion

To our knowledge, this study includes the largest cohort of SAPHO patients with mandible involvement published to date. This report provides a comprehensive description of their demographics, clinical manifestations, scintigraphic findings, and pre- and post-treatment MRI and CBCT imaging changes in the mandible.

Chronic nonbacterial inflammation of the mandible has been described using a variety of terms in addition to SAPHO syndrome. These include *primary chronic osteomyelitis* [13], *juvenile mandibular chronic osteomyelitis* (JMCO) [19], *diffuse sclerosing osteomyelitis* (DSO) [20] (suggested to represent a type of SAPHO [20, 21]), and *chronic recurrent multifocal osteomyelitis* (CRMO) [14]. In the present cohort, the author adopted the diagnosis criteria proposed by Kahn MF in an ACR meeting (2003) [17] to include isolated sterile hyperostosis/osteitis and CRMO as SAPHO syndrome.

The pathogenesis of SAPHO syndrome remains elusive, although infection with *Propionibacterium acnes* is known to be a potential trigger [17]. In our cohort, over one-third of the patients had received a dental procedure within 1 month before the onset of oral symptoms, suggesting a possible role for transient bacteraemia and tissue damage. A previous study reported the case of a woman who developed SAPHO syndrome and had mandibular involvement in the area corresponding to a tooth with recurrent caries [15]. *Prevotella*, a mucous bacterial genus found in chronic inflammatory disease, including periodontitis and rheumatoid arthritis [22], was found to regulate the inflammatory process in a mouse model of chronic multifocal osteomyelitis (CMO) (Pstpip2^cmo^ mice) [23]. The relationship between HLA-B27 and SAPHO syndrome is controversial [24], and none of our patients was positive for HLA-B27. Several other genes, such as *PSTPIP2*, *NOD2*, and *LPIN2*, have been found to be associated with SAPHO syndrome, although have yet to be confirmed pathogenic [25].

In our cohort, mandibular involvement occurred mainly in young female patients, younger than the patients without mandibular involvement. Most affected patients had only unilateral lysis of the mandible, but it can develop bilaterally over time even after decortication and partial mandible resection. Whole-body scintigraphy revealed similar proportions of axial and peripheral skeletal involvement. As reported previously [26], large joints seemed more likely than small joints to be involved.

CBCT is a commonly used three-dimensional imaging technology that requires only a low radiation dose and is performed in the stomatology department or at dental clinics; it has been specifically developed for imaging of teeth, jaws, and TMJ [27]. MRI has also been demonstrated to be a practical way to evaluate treatment effects in CRMO patients [28, 29]. As previously reported, radiographic findings in the mandible of our patients included intermingled lytic and sclerotic lesions, bone marrow oedema, periostitis and subperiosteal bone formation, and hyperostosis, similar to the lesions reported in other bones [10, 11]. Also consistent with previous studies, in our patients, medullary oedema occurred mainly on the posterior mandibular body and ramus [8], where the oedematous masseter is attached. Therefore, the notion that enthesitis could play a role in SAPHO syndrome, as has previously been suggested based on ultrasound findings [30], may require further exploration. Additionally, most of our patients exhibited cortical bone absorption combined with MRI T2 high signals in the periosteum. We therefore speculate that periostitis is also an indication of disease activity. Synovial thickening of the TMJ occurred in three patients and disappeared after treatment. Few studies have described severe destruction and ankylosis of the TMJ in SAPHO patients with mandible involvement, and conservative treatment and TMJ prosthesis grafts have both been found to be effective in functional reconstruction [8, 31].

NSAIDs are considered the first-line treatment for chronic nonbacterial osteomyelitis, with which the mean length of relapse was 3 months, similar to that of our patients who received decortication or mandible resection [9]. Aggressive therapy using TNF inhibitors and bisphosphonates could reduce disease activity and skeletal damage progression [32]. Bisphosphonates, TNF inhibitors, and methotrexate have been recommended in NSAID-resistant chronic nonbacterial osteomyelitis (CNO) or CNO with active spinal lesions [33, 34]. Bisphosphonates were found to be effective clinically and radiologically in 62–97% of patients [14, 35, 36]. None of our patients could achieve pain remission with no treatment or only NSAIDs, which prompted them to visit our hospital to seek alternative therapy. Most of our patients who received bisphosphonate, including TNF inhibitor-resistant patients, achieved remission of bone marrow oedema on MRI and bone lysis on CBCT, and the response rate was the highest among these patients. Furthermore, our patients receiving bisphosphonates with follow-up CBCT after remission did not relapse within 3 months. Severe side effects, such as osteonecrosis, have yet to be reported for bisphosphonates [37]. TNF inhibitors, including etanercept, adalimumab, and infliximab, have been reported to be effective in SAPHO patients resistant to traditional treatment [33]. TNF-α overexpression was detected in the mandible of SAPHO patients in whom etanercept was found to be effective [38]; however, not all of the mandible lesions found in our patients responded favourably to TNF inhibitors, mainly etanercept. The characteristics and management of TNF inhibitor-resistant mandible involvement in SAPHO patients therefore require further exploration. Corticosteroids are effective in controlling symptoms but appear to be unable to prevent new bone lysis in our patients. Furthermore, considering the side effects, their long-term use has not been recommended [33]. These drugs seemed to have a better effect on the involved mandible than on other osteoarticular lesions, and radiological follow-up of other lesions might be helpful. Surgery had an uncertain prognosis. In a retrospective study of 12 cases of JMCO, decortication and long-term antibiotics prevented approximately 60% of recurrences during a mean follow-up of 4.3 years [39]; however, as we found in our patients, this treatment may not prevent relapses [12, 13]. In our study, two patients underwent mandible resection with reconstruction using the fibula, and both relapsed before the first visit. Therefore, we recommend conservative treatment as the first-line management strategy in SAPHO patients with mandible involvement.

Our study has several limitations. First, most of our patients were not treatment-naïve, and multiple drugs were used in one patient because of refractory nonmandibular lesions. Thus, statistical comparison of the response to each drug was difficult. Second, not all the patients had baseline and follow-up MRI and CBCT data, which may have introduced some bias into our data. Third, because SAPHO is a chronic inflammatory disease, our follow-up period was too short to explore the long-term risk of relapse.

In conclusion, our findings suggest that mandibular involvement predominantly occurs in young patients with SAPHO syndrome. Dental procedures can precipitate its onset. CBCT imaging demonstrated mixed features of osteolytic, osteosclerotic, and hyperostotic changes. The MRI showed bone marrow and soft tissue swelling. Conservative treatment, especially intravenous bisphosphonates, can lead to significant symptomatic and radiological improvement.

## Supplementary information

**Additional file 1:** Table S1, S2, and S3.

## Data Availability

Not applicable.

## Abbreviations

SAPHO-syndrome: Synovitis, acne, pustulosis, hyperostosis, and osteitis syndrome
CBCT: Cone beam computed tomography
PPP: Palmoplantar pustulosis
SA: Severe acn
TMJ: Temporomandibular joint
MRI: Magnetic resonance imaging
VAS: Visual analogue scale
hs-CRP: High-sensitivity C-reactive protein
HLA: Human leukocyte antigen
IQR: Interquartile ranges
TwHF: *Tripterygium wilfordii* Hook F
JMCO: Juvenile mandibular chronic osteomyelitis
DSO: Diffuse sclerosing osteomyelitis
CRMO: Chronic recurrent multifocal osteomyelitis
