# Supplementary material for: Mandibular involvement in SAPHO syndrome: a retrospective study
Source: Orphanet J Rare Dis. 2020 Nov 5;15:312. doi: 10.1186/s13023-020-01589-0 (PMC7643311; doi:10.1186/s13023-020-01589-0)
Supplement: Supplementary file 1 — Additional file 1: Table S1, S2, and S3. [file 13023_2020_1589_MOESM1_ESM.docx]

**Table S1** Baseline lesions on whole-body bone scintigraphy (N=25)

| Variables | n (%) |
| --- | --- |
| Sternocostoclavicular region | 9 (36.0) |
| Ribs | 3 (12.0) |
| Axial skeleton | 8 (32.0) |
| Vertebrae | 5 (20.0) |
| Sacroiliac joints | 5 (20.0) |
| Peripheral skeleton | 9 (36.0) |
| Peripheral joints | 5 (20.0) |
| Shoulder | 1 (4.0) |
| Knee | 3 (12.0) |
| Ankle | 1 (4.0) |
| Metatarsophalangeal joints | 2 (8.0) |
| Peripheral bones‡ | 5 (20.0) |

† A total of 25 of the 26 patients (96.2%) had whole-body bone scintigraphy.

‡ Peripheral bones: involved bones included scapula, ulna, tibia, calcaneus, and humerus.

**Table S2** Treatment before the first visit to our hospital (n=26)

| Variables |  |
| --- | --- |
| Surgery intervention before baseline, n (%) | 14 (53.8) |
| Curettage/Decortication, n (%) | 12 (46.1) |
| Resection of unilateral mandible, n (%) | 2 (7.7) |
| Relapse after surgical treatment before baseline (%) | 100 |
| Duration between the surgery and relapse (months), median (range) | 2.0 (0.25-4.0) |
| Medical treatment history before baseline, n(%) | 21 (80.8) |
| NSAIDs, n(%) | 16 (61.5) |
| Glucocorticoids, n(%) | 8 (30.8) |
| Antibiotics, n(%) | 14 (53.8) |
| TNF-α inhibitors, n(%) | 5 (19.2) |
| Biphosphonates, n(%) | 1 (3.8) |
| Medical treatment in PUMCH, n(%) | 26 (100) |
| NSAIDs, n(%) | 16 (61.5) |
| Biphosphonates, n(%) | 14 (53.8) |
| Minocycline, n(%) | 10 (38.5) |
| Glucocorticoids, n(%) | 9 (34.6) |
| Tripterygium wilfordii Hook F(TII) , n(%) | 8 (30.8) |
| DMARDs, n(%) | 6 (23.1) |
| TNF-α inhibitors, n(%) | 5 (19.2) |
| JAK inhibitors, n(%) | 2 (7.7) |
| IL-6 inhibitors, n(%) | 1 (3.8) |

PUMCH: Peking Union Medical College Hospital; IQR: interquartile ranges; NSAIDs: Nonsteroidal Anti-Inflammatory Drugs; DMARDs: disease-modifying antirheumatic drugs; TNF-α inhibitors: Tumor necrosis factor alpha inhibitors; JAK inhibitors: Janus kinase inhibitors; IL-6 inhibitors: Interleukin 6 inhibitors; VAS: Visual Analogue Scale.

**Table S3** Effect of the conservative treatment in PUMCH

| Variables |  |
| --- | --- |
| Improvement in oral symptoms, n(%) | 24/26 (92.3) |
| Patients with jaw pain now, n(%) | 8/26 (30.8) |
| VAS now, median (IQR) | 1.5 (1-2.25) |
| Patients with face swelling now, n(%) | 1/22 (4.5) |
| Patients with limitation of mouth opening now, n/N (%) | 2/20 (10.0) |
| Improvement in other osteoarticular symptoms, n/N (%) | 3/8 (37.5) |
| Improvement in dermatological symptoms, n/N (%) | 2/15 (13.3) |

VAS: Visual Analogue Scale; IQR: interquartile ranges.
